# Seropositivity and Antigen-Specific Seroreactivity to *Leptospira* spp. In Equids from Three Regions of Veracruz, Mexico

**DOI:** 10.3390/ani16193130

**Published:** 2026-10-06

**Authors:** Alejandro Taylor Estrada-Coates, Juan Manuel Pinos-Rodríguez, Jorge Genaro Vicente-Martínez, Isaac De Gasperin-López, Jessica Yamilete Rodríguez-Flores, Miriam Alva-Trujillo

**Affiliations:** Facultad de Medicina Veterinaria y Zootecnia, Universidad Veracruzana, Miguel Ángel de Quevedo and Agustín Yáñez, Unidad Veracruzana, Veracruz 91710, Mexico; aestrada@uv.mx (A.T.E.-C.); jpinos@uv.mx (J.M.P.-R.); idegasperin@uv.mx (I.D.G.-L.); jessrodriguez@uv.mx (J.Y.R.-F.); mialva@uv.mx (M.A.-T.)

**Keywords:** equids, microscopic agglutination test, serology, epidemiology, antibodies, zoonosis

## Abstract

Information on *Leptospira* spp. exposure in Mexican equids remains limited. This cross-sectional study evaluated seropositivity and antigen-specific seroreactivity in 313 apparently healthy equids enrolled by convenience sampling in Los Tuxtlas (*n* = 119), Sotavento (*n* = 108), and Capital (*n* = 86), Veracruz, Mexico, from 2 October 2023 to 6 March 2024. Sera were examined by the microscopic agglutination test (MAT) against 13 antigens; titers ≥ 1:100 defined seropositivity. Overall seropositivity was 57.5% (180/313; 95% confidence interval [CI]: 52.0–62.9), with regional proportions of 49.6%, 65.7%, and 58.1%, respectively (unadjusted *p* = 0.048). Modified Poisson models with robust variance were fitted separately for region with species and region with sex. For Sotavento versus Los Tuxtlas, the adjusted prevalence ratio was 1.33 after adjustment for species (95% CI: 1.06–1.67) and 1.33 after adjustment for sex (95% CI: 1.06–1.66). Antigen-specific seroreactivity was most frequent for Australis (63/313; 20.1%), Bratislava (62/313; 19.8%), and Pyrogenes (57/313; 18.2%); these are overlapping antigen responses, not mutually exclusive serogroup estimates. Environmental and management exposures were not measured at the animal level. The findings support surveillance at the animal–human–environment interface but do not demonstrate active shedding or zoonotic transmission.

## Simple Summary

Equids play an important role in many rural activities and may be exposed to bacteria of the genus *Leptospira* through infected animals and contaminated environments. In Mexico, information on this exposure in equid populations remains limited. In this study, 313 horses, donkeys, and hybrids from three regions of Veracruz, Mexico, were evaluated. More than half of the animals had antibodies against *Leptospira*, and the proportion of seropositive animals varied among regions. Regional differences were also observed in antigen-specific reactivity, whereas equid species and sex were not associated with seropositivity. Because individual animals could react to more than one antigen, antigen-specific results were summarized at the animal level. These findings show that exposure to *Leptospira* was frequent and geographically heterogeneous among the equids studied. The results expand current knowledge of *Leptospira* exposure in equid populations from Veracruz, Mexico, and may help inform animal-health surveillance.

## 1. Introduction

Leptospirosis is a globally distributed bacterial zoonosis caused by pathogenic species of the genus *Leptospira*. Its epidemiology involves numerous domestic and wild hosts, as well as contamination of water and soil, which may facilitate indirect exposure and contribute to the persistence of the pathogen in different environments [1,2,3,4].

In equids, exposure to *Leptospira* spp. may occur without evident clinical signs; however, infection has also been associated with recurrent uveitis, reproductive disorders, and renal disease [5,6,7]. Available epidemiological evidence indicates a broad distribution of anti-*Leptospira* antibodies in equine populations. A recent meta-analysis of 35 cross-sectional studies published between 2015 and 2025 estimated a pooled global seroprevalence of 47.2% (95% confidence interval [CI]: 37.6–57.1), with substantial heterogeneity among studies and regions [8].

Equids, including horses, donkeys, and their hybrids, contribute to agriculture, transport, and rural livelihoods in many settings [9]. However, the specific work performed by individual animals in the present study was not recorded; therefore, the sampled population is referred to simply as equids. These populations remain epidemiologically relevant because of their interaction with rural environments and other animal species, and serological surveys can contribute to animal-health surveillance.

In Mexico, contemporary information on exposure of equids to *Leptospira* spp. remains limited. A seropositivity of 77.8% was documented in donkeys from Durango, with variation associated with municipality and establishment of origin [10]. More recently, *Leptospira interrogans* and *Leptospira santarosai* were confirmed in equids from Veracruz by bacteriological isolation and molecular characterization, providing direct evidence of pathogenic leptospires in the state [11]. Nevertheless, the regional distribution of seropositivity and patterns of seroreactivity in equid populations from Veracruz remain poorly characterized.

Recent microbiological evidence from Veracruz confirms the local occurrence of pathogenic leptospires but does not establish how antibody detection is distributed among equids in different regions [11]. The present study therefore aimed to estimate the proportion of sampled equids seropositive to *Leptospira* spp. and to describe antigen-specific seroreactivity in Los Tuxtlas, Sotavento, and Capital, Veracruz, Mexico. The working hypothesis anticipated higher seroreactivity in Los Tuxtlas than in the other two regions. Associations with region, species, and sex were evaluated within the limitations of the available data.

## 2. Materials and Methods

### 2.1. Study Design and Population

A cross-sectional epidemiological study was conducted from 2 October 2023 to 6 March 2024 in equids from three regions of Veracruz, Mexico: Los Tuxtlas, Sotavento, and Capital. Animals were enrolled by convenience sampling with the voluntary participation of their owners. The sampling window began near the end of the usual rainy period and continued through generally drier months in Veracruz; however, individual sampling dates were not retained in linkage with serological results, so month- or season-specific analyses were not possible.

A total of 313 equids were evaluated: 119 from Los Tuxtlas, 108 from Sotavento, and 86 from Capital. The study population comprised 175 horses, 123 donkeys, and 15 hybrids. Animals of both sexes, at least six months of age and apparently healthy at the time of sampling, were included. A general physical examination was performed before sample collection. Mean age was 8.8 ± 5.6 years (range: 6 months–25 years) in Los Tuxtlas, 9.2 ± 6.2 years (range: 8 months–22 years) in Sotavento, and 7.9 ± 5.9 years (range: 9 months–28 years) in Capital; the combined mean was 8.7 ± 5.9 years (range: 6 months–28 years). For epidemiological description, age was categorized as 6 months–<1 year, 1–5 years, and >5 years.

The study sample comprised animals available through participating owners. No a priori sample-size calculation for regional comparisons was documented; therefore, the final sample reflected animal availability and owner participation. Seropositivity is reported for the animals examined and is not presented as a population-representative seroprevalence estimate for all equids in Veracruz. Workload, husbandry practices, housing, animal density, water sources, flooding history, and contact with cattle, dogs, rodents, or other potential reservoirs were not systematically recorded and could not be analyzed as risk factors. At the time of the study, no vaccine against equine leptospirosis was commercially available in Mexico; accordingly, no vaccinated subgroup was analyzed.

### 2.2. Study Area

The Los Tuxtlas, Sotavento, and Capital regions are located in the state of Veracruz in eastern Mexico and were defined according to the official regional planning framework of the Government of Veracruz [12]. Los Tuxtlas covers approximately 2947 km^2^ and includes Catemaco, Hueyapan de Ocampo, San Andrés Tuxtla, and Santiago Tuxtla; Sotavento covers approximately 3961 km^2^ and includes 12 municipalities; and Capital covers approximately 5327 km^2^ and includes 33 municipalities [12]. Historical regional descriptions characterize Los Tuxtlas as predominantly warm and very humid, with temperatures of approximately 22–26 °C and annual precipitation of about 1600–2500 mm, locally reaching approximately 4500 mm. The broader Sotavento coastal lowlands are warm, with temperatures of approximately 22–27 °C and annual precipitation of about 800–2500 mm [13]. Capital extends from lower-elevation areas to foothills and highlands and therefore encompasses substantial altitudinal and climatic heterogeneity [12].

These quantitative values are historical regional descriptors rather than measurements from the 2023–2024 sampling period at individual holdings. Sampling-site temperature, precipitation, and relative humidity were not linked to individual animals, and comparable regional relative-humidity data for the sampling period were not available in the retained records. Likewise, flooding exposure, livestock density, water management, rodent occurrence, and contact with other domestic or wild reservoirs were not measured at the animal or holding level. Region was therefore used as a geographic descriptor and not as a direct proxy for environmental exposure. The geographic context of the three study regions is shown in Figure 1.

### 2.3. Sample Collection, Processing, and Storage

Blood samples were collected by aseptic jugular venipuncture using 20 G × 25 mm BD needles into 7 mL evacuated tubes without anticoagulant (BD Vacutainer^®^; Becton, Dickinson and Company, Franklin Lakes, NJ, USA).

Samples were centrifuged at 1500× *g* for 15 min using a Hettich EBA 200 centrifuge (Andreas Hettich GmbH & Co. KG, Tuttlingen, Germany). The resulting serum was transferred to 2.0 mL microtubes (Eppendorf SE, Hamburg, Germany) in 0.5 mL aliquots. Aliquots were maintained at approximately 4 °C during transport in insulated coolers with refrigerated gel packs and subsequently stored at −20 °C until shipment for serological analysis.

### 2.4. Microscopic Agglutination Test

Serum samples were analyzed using the microscopic agglutination test (MAT) at the Centro Nacional de Servicios de Diagnóstico en Salud Animal (CENASA), Servicio Nacional de Sanidad, Inocuidad y Calidad Agroalimentaria (SENASICA), Tecámac, State of Mexico, Mexico. The laboratory panel comprised 13 live *Leptospira* antigens: Hardjo, Grippotyphosa, Pyrogenes, Tarassovi, Bratislava, Australis, Shermani, Hebdomadis, Pomona, Canicola, Icterohaemorrhagiae, Wolffi, and Ballum. These names identify test antigens rather than 13 independent serogroups. Australis and Bratislava are distinct serovar antigens within serogroup Australis, whereas Hardjo and Wolffi belong to serogroup Sejroe. Responses to antigens within the same serogroup were therefore not summed to estimate serogroup-specific numbers of positive animals. The MAT used the CENASA live-antigen panel rather than a commercial diagnostic kit; consequently, commercial kit catalogue numbers were not applicable.

Reactive sera were titrated using twofold serial dilutions of 1:100, 1:200, 1:400, 1:800, and 1:1600. The available laboratory record identifies 1:100 as the lowest dilution evaluated and as the seropositivity threshold. The titer was defined as the highest dilution showing at least 50% agglutination relative to the corresponding antigen control. An animal was considered seropositive when a titer ≥ 1:100 was detected against at least one antigen in the panel. Reactions were read using an Olympus BX43 microscope (Olympus Corporation, Tokyo, Japan). MAT interpretation followed the principles described by the World Organisation for Animal Health [14,15,16]. The retained study records did not specify whether samples were tested in duplicate or identify separate positive and negative serum controls; these details therefore cannot be reported without the original laboratory bench records.

Because an individual animal could react to more than one antigen, overall seropositivity and antigen-specific seroreactivity were treated as distinct measures. Overall seropositivity counted each animal only once. For each antigen, the numerator was the number of animals with a reactive result and the denominator was the number of animals examined in the corresponding region. Thus, an animal could contribute to several antigen-specific summaries, but no analysis treated the pooled 349 positive animal–antigen results as 349 independent subjects.

### 2.5. Statistical Analysis

Categorical variables were summarized as counts and percentages. Wilson 95% confidence intervals (CIs) were calculated for overall, subgroup, and antigen-specific seropositivity proportions. Pearson’s chi-square tests without continuity correction were used for univariable comparisons by region, species, sex, and age group. Pairwise regional comparisons used the same test with Holm adjustment across the three comparisons. Antigen-specific summaries were descriptive; no joint inferential comparison treated multiple antigen responses from the same animal as independent. Supplementary multivariable analyses used the available region-by-species-by-serostatus and region-by-sex-by-serostatus cell frequencies. Modified Poisson generalized linear models with a log link and HC0 robust sandwich variance were fitted to estimate adjusted prevalence ratios (aPRs) [17]. Two models were fitted because the retained tabulations did not preserve the joint species-by-sex distribution within region and serostatus: Model 1 included region and species, and Model 2 included region and sex. Los Tuxtlas, horses, and females were the reference categories. Age could not be incorporated into these regional models because the required joint tabulation was unavailable. Statistical significance was set at *p* < 0.05. Analyses were performed using Python 3.13.5, SciPy 1.17.0, and statsmodels 0.14.6.

### 2.6. Ethical Considerations

The study was approved by the Animal Well-being Committee of the Facultad de Medicina Veterinaria y Zootecnia, Universidad Veracruzana (approval No. BA2023-05; 5 May 2023). Verbal informed consent was obtained from all owners prior to the enrollment of their animals in the study and sample collection.

## 3. Results

### 3.1. Overall Seropositivity and Regional Distribution

Serum samples from 313 equids were analyzed. Of the animals evaluated, 180 had MAT titers ≥ 1:100 against at least one antigen in the panel, corresponding to an overall seropositivity of 57.5% (95% CI: 52.0–62.9).

Seropositivity differed among the study regions (χ^2^ = 6.071; *df* = 2; *p* = 0.048). Sotavento had the highest frequency at 65.7% (71/108), followed by Capital at 58.1% (50/86) and Los Tuxtlas at 49.6% (59/119). In pairwise comparisons adjusted using the Holm procedure, seropositivity was higher in Sotavento than in Los Tuxtlas (adjusted *p* = 0.042), whereas Capital did not differ from either Los Tuxtlas or Sotavento (adjusted *p* = 0.451 for both comparisons). The regional distribution of seropositivity is summarized in Table 1.

### 3.2. Seropositivity by Species, Sex, and Age

Seropositivity was 57.7% (101/175; 95% CI: 50.3–64.8) in horses, 58.5% (72/123; 95% CI: 49.7–66.9) in donkeys, and 46.7% (7/15; 95% CI: 24.8–69.9) in hybrids. No association was observed between species and seropositivity (χ^2^ = 0.778; *df* = 2; *p* = 0.678).

By sex, seropositivity was 58.0% (80/138; 95% CI: 49.6–65.9) in females and 57.1% (100/175; 95% CI: 49.7–64.2) in males. Sex was not associated with seropositivity (χ^2^ = 0.022; *df* = 1; *p* = 0.883). Seropositivity according to species and sex is presented in Table 2.

Seropositivity increased across the three age groups: 28.6% (16/56; 95% CI: 18.4–41.5) in animals aged 6 months–<1 year, 54.7% (52/95; 95% CI: 44.7–64.4) in those aged 1–5 years, and 69.1% (112/162; 95% CI: 61.6–75.7) in animals aged >5 years (χ^2^ = 28.451; *df* = 2; *p* < 0.001). Because individual sampling dates were not linked to serological records, month-wise and season-wise seropositivity could not be estimated.

The available regional tabulation also allowed species and sex composition and seropositivity to be described by region (Table 3). The 15 hybrids were represented in all three regions: five in Los Tuxtlas, six in Sotavento, and four in Capital.

### 3.3. Regional Associations Adjusted for Species or Sex

In Model 1, which adjusted region for species, the aPR for Sotavento versus Los Tuxtlas was 1.33 (95% CI: 1.06–1.67; *p* = 0.014), and the aPR for Capital versus Los Tuxtlas was 1.17 (95% CI: 0.91–1.51; *p* = 0.219). Donkeys did not differ from horses (aPR = 0.99; 95% CI: 0.82–1.21; *p* = 0.944), and the hybrid estimate was imprecise (aPR = 0.79; 95% CI: 0.45–1.37; *p* = 0.403). In Model 2, which adjusted region for sex, the aPR for Sotavento versus Los Tuxtlas was 1.33 (95% CI: 1.06–1.66; *p* = 0.015), the aPR for Capital versus Los Tuxtlas was 1.17 (95% CI: 0.91–1.52; *p* = 0.223), and the male-versus-female aPR was 1.00 (95% CI: 0.82–1.21; *p* = 0.987). These are separate adjustments rather than one model simultaneously controlling for species and sex. The results of both models are summarized in Table 4.

### 3.4. Antigen-Specific Seroreactivity

The 180 seropositive animals produced 349 positive animal–antigen results because some animals reacted to more than one antigen. These are overlapping responses from the same animals, not 349 independent subjects. Antigen-specific numerators and denominators are presented in Table 5.

Across all 313 animals, antigen-specific seroreactivity was most frequent for Australis (63/313; 20.1%), followed by Bratislava (62/313; 19.8%), Pyrogenes (57/313; 18.2%), and Tarassovi (35/313; 11.2%). Australis and Bratislava are separate test antigens within serogroup Australis and were not combined; similarly, Hardjo and Wolffi belong to serogroup Sejroe. These overlapping antigen-specific proportions must not be summed to estimate the proportion reactive to a combined serogroup.

Descriptively, Pyrogenes had the highest antigen-specific proportion in Los Tuxtlas (21/119; 17.6%), whereas Bratislava had the highest proportion in Sotavento (33/108; 30.6%) and Capital (23/86; 26.7%). At least one animal reacted to each of the 13 antigens in the combined sample, but not in every region: Pomona had no reactive animals in Sotavento, and Ballum had no reactive animals in Sotavento or Capital. The only Ballum-reactive animal was from Los Tuxtlas (1/119; 0.8%). These rankings are descriptive and are not presented as an inferential comparison of multivariate regional antigen profiles.

### 3.5. Distribution of MAT Titers

Titers for the 349 MAT reactions ranged from 1:100 to 1:1600. A titer of 1:100 was most frequent, accounting for 225 reactions (64.5%), followed by 1:200 with 74 (21.2%), 1:400 with 26 (7.4%), 1:800 with 17 (4.9%), and 1:1600 with 7 (2.0%). Overall, 85.7% of reactions had titers of 1:100 or 1:200. This is a descriptive distribution of positive animal–antigen results, not of each animal’s highest titer; no inferential tests were applied to the pooled reaction counts.

## 4. Discussion

The magnitude of seropositivity observed is consistent with the substantial exposure documented in equine populations from different regions. The 2026 meta-analysis estimated a pooled global seroprevalence of 47.2% and a pooled estimate of 55.0% for South America [8]. In Goiás, Brazil, seropositivity of 61.6% was documented among 894 equids [18], whereas 45.9% was reported in horses from southern Brazil [19]. These comparisons place the 57.5% observed in Veracruz within the broad range reported for equine populations; however, differences in sampling design, antigen panel, cutoff titer, and population characteristics should be considered when comparing estimates across studies. Studies from Europe, Africa, and the Americas likewise show considerable variation in seropositivity according to the population, epidemiological setting, antigen panel, and cutoff titer [20,21,22,23,24,25,26,27,28].

The findings are particularly relevant in the Mexican context. High seropositivity and geographic variation were documented in donkeys from Durango [10]. Recent bacteriological isolation and molecular characterization of *L. interrogans* and *L. santarosai* in equids from Veracruz [11] provide direct microbiological support for the present serological findings and complement the serological characterization of 313 equids distributed across three regions of the state.

The higher seropositivity observed in Sotavento than in Los Tuxtlas was similar after separate adjustment for species and sex: the Sotavento-versus-Los Tuxtlas aPR was 1.33 in both models, with overlapping 95% CIs. These models do not establish an independent regional effect after simultaneous control for species, sex, age, season, husbandry, or environmental conditions because the required joint data were not available. Spatial and seasonal variation has been reported in northeastern Brazil [29], and environmental or management associations have been investigated in South African and Ethiopian equids [23,24]. These studies support investigation of explanatory exposures rather than attribution of the present regional differences to unmeasured conditions.

Historical regional context indicates environmental contrasts within Veracruz: Los Tuxtlas is generally warmer and wetter, Sotavento comprises warm coastal lowlands, and Capital spans marked altitudinal and climatic gradients [12,13]. These descriptors are biologically relevant background but were not measurements of the conditions experienced by each sampled animal. Sampling-period temperature, precipitation, relative humidity, flooding, livestock density, water management, and rodent ecology were not recorded at the animal or holding level; therefore, they cannot be used to explain the regional seropositivity differences observed here.

Antigen-specific seroreactivity varied descriptively among regions. Pyrogenes had the highest animal-level proportion in Los Tuxtlas, whereas Bratislava had the highest proportion in Sotavento and Capital. The 2026 global meta-analysis identified serogroup Australis as frequently reported in equids and Bratislava as a commonly reported serovar [8,30]. In the present MAT panel, however, Australis and Bratislava were separate test antigens within the same serogroup and were not summed. The same distinction applies to Hardjo and Wolffi within serogroup Sejroe. Accordingly, the present data describe responses to test antigens and do not identify infecting serovars or combined serogroup-specific prevalence.

The 180 seropositive animals yielded 349 positive animal–antigen results because some animals reacted to multiple antigens. Multiple MAT responses can reflect cross-reactivity and do not demonstrate infection with multiple serovars [14,15,19,31]. Presenting each antigen against the total number of animals examined prevents animals with more reactive antigens from receiving greater weight within a given antigen-specific proportion. Nevertheless, antigen responses within the same animal remain correlated. Because the individual antigen-response matrix was not retained, a joint model accounting for that correlation could not be fitted; regional antigen rankings are therefore descriptive. Pomona, Ballum, and Icterohaemorrhagiae had zero observations in one or more regions, and Ballum was detected in only one animal; zero observed counts should not be interpreted as proof of absence of exposure in the underlying regional population.

Most reactions had titers of 1:100 or 1:200, which together accounted for 85.7% of all reactions, whereas titers of 1:800 and 1:1600 were considerably less frequent. The magnitude of a titer obtained from a single sample cannot, by itself, establish the timing of exposure or define a specific clinical status. MAT results should be interpreted in light of the epidemiological context and antigen panel used and, when clinical disease is investigated, paired samples or complementary diagnostic techniques may be warranted [14,15,16].

Species was not associated with seropositivity. Horses and donkeys had nearly identical frequencies, whereas the estimate for hybrids was less precise because only 15 animals were included. All 15 hybrids were nevertheless represented across the three regions (five in Los Tuxtlas, six in Sotavento, and four in Capital). Likewise, females and males had similar frequencies and no sex association was detected. In contrast, seropositivity increased across the three age groups in univariable analysis, from 28.6% in animals aged 6 months–<1 year to 69.1% in those aged >5 years. Age could not be incorporated into the regional adjusted models because the required joint tabulation was unavailable.

All animals were considered apparently healthy at the time of sampling, showing that seroreactivity to *Leptospira* can be frequent in equine populations without evident clinical signs. This finding is consistent with a Colorado study in which 82% of 124 apparently healthy equids had MAT titers ≥1:100 [32]. The absence of clinical signs at sampling does not eliminate the health relevance of exposure because equine leptospirosis has been associated with recurrent uveitis, reproductive disorders, and renal involvement [5,32,33,34,35].

The present investigation was designed as a serological survey, and only blood/serum specimens were collected. Urine, tissue, or other specimens intended for direct organism detection were not obtained; therefore, culture and polymerase chain reaction (PCR) were not performed. Consequently, the study cannot establish current infection, identify the infecting species or serovar, or demonstrate urinary shedding. Complementary organism-detection methods and, where clinically indicated, paired serum samples would be required to address those questions [14,15,16,33,35].

The public-health relevance of these findings lies in the possibility of shared exposure of animals and people to contaminated environments, not in demonstrated equid-to-human transmission. Studies involving equids and occupationally exposed people support investigation of these interfaces [36,37,38], while microbiological studies show that some equids can harbor pathogenic leptospires [33,35]. In rural communities, prudent preventive measures include protecting feed and drinking-water sources from animal urine, reducing rodent access to stored feed, improving drainage where feasible, and using appropriate protection when handling urine or contaminated materials. Veterinarians should consider leptospirosis in compatible clinical presentations and use complementary diagnostics when active infection or shedding is suspected.

The principal limitations are convenience sampling, absence of individual environmental and management risk-factor data, incomplete joint covariate information, and the cross-sectional serological design. Voluntary owner participation may introduce selection bias, so the observed proportions should not be interpreted as population-representative regional seroprevalence estimates. Sampling month, workload, husbandry, housing, water sources, flooding, livestock density, contact with other animals, and rodent occurrence were not available as individual covariates. Sampling dates were not linked to serological records, preventing month- or season-specific analysis. At the time of the study, no equine leptospirosis vaccine was commercially available in Mexico. Owner- or holding-level clustering could not be accounted for because cluster identifiers were unavailable, and no a priori sample-size calculation for regional comparisons was documented. These limitations constrain generalization and causal interpretation of regional differences.

Two strengths of the study were the inclusion of horses, donkeys, and hybrids and the availability of responses to all 13 MAT antigens. Future studies should retain individual identifiers linking full MAT response profiles, age, sampling date, region, species, sex, owner or holding, and measured environmental and management exposures. Prespecified sampling and analysis plans would permit simultaneous multivariable adjustment, cluster-aware uncertainty estimates, seasonal analyses, and complementary investigation of active infection.

## 5. Conclusions

Seropositivity to *Leptospira* spp. was frequent among the sampled equids. The higher proportion observed in Sotavento than in Los Tuxtlas remained similar after separate adjustment for species and sex, although the available data did not permit one model simultaneously controlling for both covariates, age, season, management, or environmental exposures. Species and sex were not associated with seropositivity in the available analyses, whereas seropositivity increased across age groups in univariable analysis. Antigen-specific responses were described at the animal level and should not be interpreted as identified infecting serovars or independent regional multivariate profiles. Convenience sampling and incomplete exposure information limit generalization and causal interpretation.

Further studies should combine documented sampling, individual-level exposure data, and complementary microbiological or molecular testing. Veterinary surveillance, protection of feed and water from contamination, rodent control around stored feed, and safe handling of urine or contaminated materials are appropriate practical priorities for veterinarians and rural communities. Seropositivity alone does not demonstrate active shedding or zoonotic transmission.

## Figures and Tables

**Figure 1:**
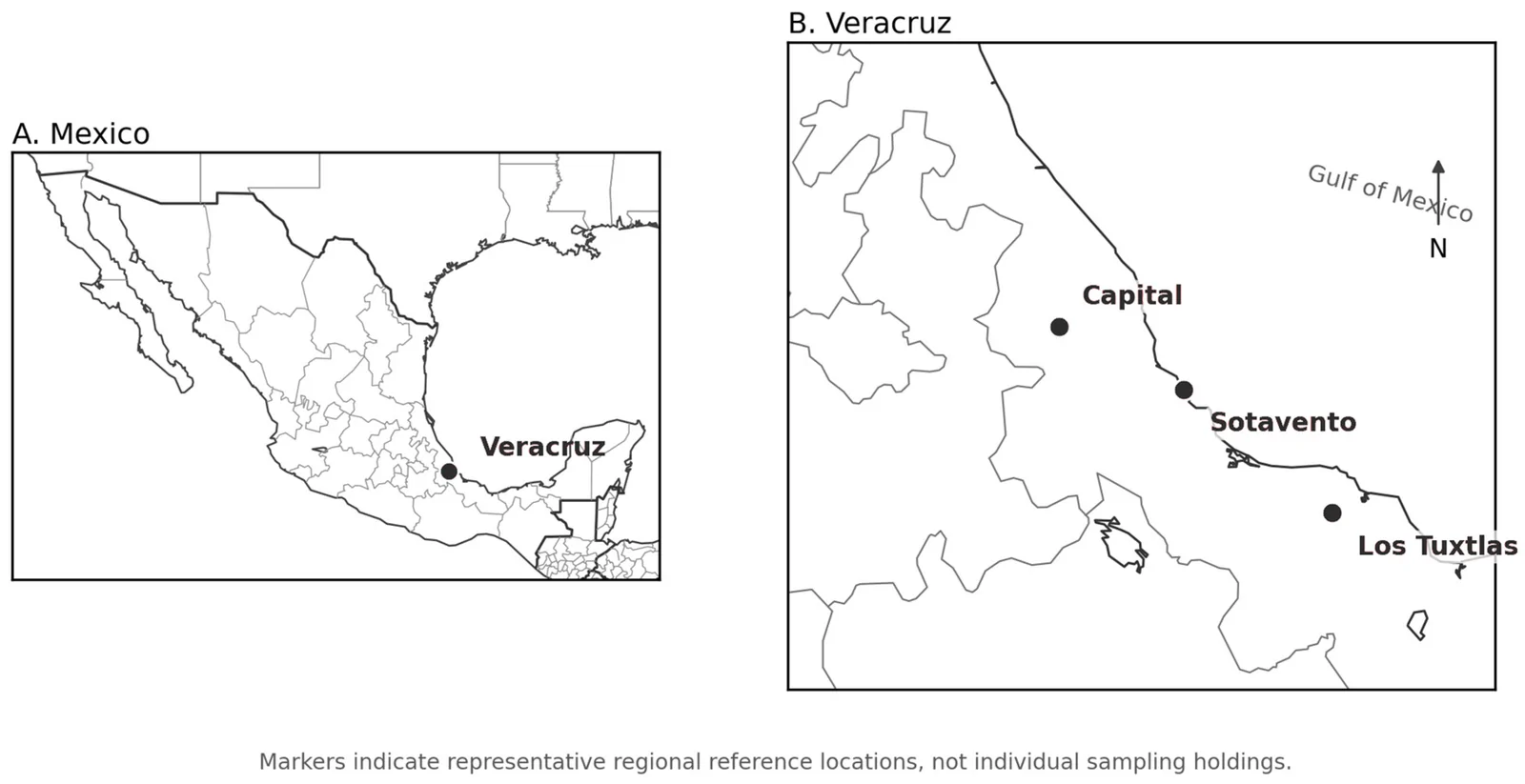
Geographic context of the three study regions in Veracruz, Mexico. Markers indicate representative regional reference locations for Capital, Sotavento, and Los Tuxtlas and do not identify individual sampling holdings. Regional nomenclature follows the official planning framework of the Government of Veracruz [12].

**Table 1:** Seropositivity to *Leptospira* spp. in equids from three regions of Veracruz, Mexico.

Region	Examined, *n*	Seropositive, *n*	Seronegative, *n*	Seropositivity, % (95% CI)
Los Tuxtlas	119	59	60	49.6 (40.8–58.4)
Sotavento	108	71	37	65.7 (56.4–74.0)
Capital	86	50	36	58.1 (47.6–68.0)
Total	313	180	133	57.5 (52.0–62.9)

CI, confidence interval; MAT, microscopic agglutination test. An animal was considered seropositive when a MAT titer ≥ 1:100 was detected against at least one antigen evaluated.

**Table 2:** Seropositivity to *Leptospira* spp. according to species, sex, and age group of the equids studied.

Variable	Category	Examined, *n*	Seropositive, *n*	Seronegative, *n*	Seropositivity, % (95% CI)
Species	Horses	175	101	74	57.7 (50.3–64.8)
	Donkeys	123	72	51	58.5 (49.7–66.9)
	Hybrids	15	7	8	46.7 (24.8–69.9)
Sex	Females	138	80	58	58.0 (49.6–65.9)
	Males	175	100	75	57.1 (49.7–64.2)
Age group	6 months–<1 year	56	16	40	28.6 (18.4–41.5)
	1–5 years	95	52	43	54.7 (44.7–64.4)
	>5 years	162	112	50	69.1 (61.6–75.7)

CI, confidence interval. Species: χ^2^ = 0.778; *df* = 2; *p* = 0.678. Sex: χ^2^ = 0.022; *df* = 1; *p* = 0.883. Age group: χ^2^ = 28.451; *df* = 2; *p* < 0.001. Age-group analysis was univariable.

**Table 3:** Regional distribution and seropositivity according to species and sex.

Variable	Category	Los Tuxtlas *n*; + (%)	Sotavento *n*; + (%)	Capital *n*; + (%)	Overall *n*; + (%)
Species	Horses	70; 34 (48.6)	52; 36 (69.2)	53; 31 (58.5)	175; 101 (57.7)
	Donkeys	44; 23 (52.3)	50; 32 (64.0)	29; 17 (58.6)	123; 72 (58.5)
	Hybrids	5; 2 (40.0)	6; 3 (50.0)	4; 2 (50.0)	15; 7 (46.7)
Sex	Females	55; 27 (49.1)	58; 37 (63.8)	25; 16 (64.0)	138; 80 (58.0)
	Males	64; 32 (50.0)	50; 34 (68.0)	61; 34 (55.7)	175; 100 (57.1)

Each cell reports examined *n*; seropositive *n* (seropositivity %). Percentages use the corresponding region–category stratum as the denominator. The overall column uses the full category total. MAT, microscopic agglutination test.

**Table 4:** Adjusted prevalence ratios from separate models including region with species or sex.

Model	Comparison	aPR	95% CI	*p*-Value
Region + species	Sotavento vs. Los Tuxtlas	1.33	1.06–1.67	0.014
Region + species	Capital vs. Los Tuxtlas	1.17	0.91–1.51	0.219
Region + species	Donkeys vs. horses	0.99	0.82–1.21	0.944
Region + species	Hybrids vs. horses	0.79	0.45–1.37	0.403
Region + sex	Sotavento vs. Los Tuxtlas	1.33	1.06–1.66	0.015
Region + sex	Capital vs. Los Tuxtlas	1.17	0.91–1.52	0.223
Region + sex	Males vs. females	1.00	0.82–1.21	0.987

aPR, adjusted prevalence ratio; CI, confidence interval. Modified Poisson models used a log link and HC0 robust sandwich variance. Model 1 includes region and species; Model 2 includes region and sex. Los Tuxtlas, horses, and females are the corresponding reference categories. The retained tabulations did not permit one model including both species and sex simultaneously; neither model controls for age, season, management, environmental exposures, or holding-level clustering.

**Table 5:** Antigen-specific seroreactivity to 13 *Leptospira* antigens by region.

MAT Antigen	Los Tuxtlas (*n* = 119)	Sotavento (*n* = 108)	Capital (*n* = 86)	Overall (*n* = 313)
Hardjo	7; 5.9 (2.9–11.6)	10; 9.3 (5.1–16.2)	3; 3.5 (1.2–9.8)	20; 6.4 (4.2–9.7)
Grippotyphosa	11; 9.2 (5.2–15.8)	7; 6.5 (3.2–12.8)	14; 16.3 (10.0–25.5)	32; 10.2 (7.3–14.1)
Pyrogenes	21; 17.6 (11.8–25.5)	20; 18.5 (12.3–26.9)	16; 18.6 (11.8–28.1)	57; 18.2 (14.3–22.9)
Tarassovi	15; 12.6 (7.8–19.8)	9; 8.3 (4.4–15.1)	11; 12.8 (7.3–21.5)	35; 11.2 (8.2–15.2)
Bratislava	6; 5.0 (2.3–10.6)	33; 30.6 (22.7–39.8)	23; 26.7 (18.5–36.9)	62; 19.8 (15.8–24.6)
Australis	16; 13.4 (8.4–20.7)	31; 28.7 (21.0–37.9)	16; 18.6 (11.8–28.1)	63; 20.1 (16.1–24.9)
Shermani	4; 3.4 (1.3–8.3)	8; 7.4 (3.8–13.9)	4; 4.7 (1.8–11.4)	16; 5.1 (3.2–8.1)
Hebdomadis	5; 4.2 (1.8–9.5)	2; 1.9 (0.5–6.5)	5; 5.8 (2.5–12.9)	12; 3.8 (2.2–6.6)
Pomona	1; 0.8 (0.1–4.6)	0; 0.0 (0.0–3.4)	3; 3.5 (1.2–9.8)	4; 1.3 (0.5–3.2)
Canicola	3; 2.5 (0.9–7.2)	17; 15.7 (10.1–23.8)	4; 4.7 (1.8–11.4)	24; 7.7 (5.2–11.2)
Icterohaemorrhagiae	2; 1.7 (0.5–5.9)	10; 9.3 (5.1–16.2)	2; 2.3 (0.6–8.1)	14; 4.5 (2.7–7.4)
Wolffi	6; 5.0 (2.3–10.6)	2; 1.9 (0.5–6.5)	1; 1.2 (0.2–6.3)	9; 2.9 (1.5–5.4)
Ballum	1; 0.8 (0.1–4.6)	0; 0.0 (0.0–3.4)	0; 0.0 (0.0–4.3)	1; 0.3 (0.1–1.8)
Total	98 (100)	149 (100)	102 (100)	349 (100)

MAT, microscopic agglutination test; CI, confidence interval. Each cell shows reactive-animal count; percentage (Wilson 95% CI). Denominators are all animals examined in each region (119, 108, and 86) or overall (313), not numbers of positive reactions. Each animal contributes at most once to a given antigen but may appear in several rows; percentages therefore need not sum to 100%. Australis and Bratislava belong to serogroup Australis; Hardjo and Wolffi belong to serogroup Sejroe. These are antigen-specific responses, not identified infecting serovars or summed serogroup frequencies.

## Data Availability

The data supporting the findings of this study are available from the corresponding author upon reasonable request.

## References

[1] Vincent A.T., Schiettekatte O., Goarant C., Neela V.K., Bernet E., Thibeaux R., Ismail N., Mohd Khalid M.K.N., Amran F., Masuzawa T. (2019). Revisiting the taxonomy and evolution of pathogenicity of the genus *Leptospira* through the prism of genomics. PLoS Negl. Trop. Dis..

[2] Sayanthi Y., Susanna D. (2024). Pathogenic *Leptospira* contamination in the environment: A systematic review. Infect. Ecol. Epidemiol..

[3] Adler B., de la Peña Moctezuma A. (2010). *Leptospira* and leptospirosis. Vet. Microbiol..

[4] Ellis W.A. (2015). Animal leptospirosis. Curr. Top. Microbiol. Immunol..

[5] Divers T.J., Chang Y.-F., Irby N.L., Smith J.L., Carter C.N. (2019). Leptospirosis: An important infectious disease in North American horses. Equine Vet. J..

[6] Díaz E.A., Arroyo G., Sáenz C., Mena L., Barragán V. (2023). Leptospirosis in horses: Sentinels for a neglected zoonosis? A systematic review. Vet. World.

[7] Toriz-Suarez O., Pérez-Rivero J., Herrera-Barragán A., Torres-Barranca J., Lombardero-Goldaracena G. (2021). Frecuencia de leptospirosis en equinos: Revisión de literatura. Abanico Vet..

[8] Concha D., Azócar-Aedo L., Ricardo T., Rodríguez A., Ramírez R., Gallardo Paffetti M. (2026). Epidemiological evidence of anti-*Leptospira* antibodies in horses at a global level (years 2015 to 2025): A systematic review and meta-analysis. J. Equine Vet. Sci..

[9] Raw Z., Collins J.A., Burden F.A. (2024). What Is a Working Equid? Analysis of Current Terminology and a Suggested Definition. Animals.

[10] Alvarado-Esquivel C., Cruz-Romero A., Romero-Salas D., Alvarado-Félix Á.O., Aguilar-Domínguez M., Ochoa-Valencia J.L., Alvarado-Esquivel D., Hernández-Tinoco J., Zamarripa-Barboza J.A., Sánchez-Anguiano L.F. (2018). Apparently high *Leptospira* antibody seropositivity in donkeys for slaughter in three municipalities in Durango, Mexico. J. Vet. Diagn. Investig..

[11] Ramos-Vázquez J.R., Sánchez-Montes S., Esparza-González S.C., Romero-Salas D., Pardio-Sedas V., Estrada-Coates A.T., Alva-Trujillo M., Ballados-González G.G., Grostieta E., Becker I. (2024). Isolation and molecular identification of *Leptospira santarosai* and *Leptospira interrogans* in equines from eastern Mexico. Acta Trop..

[12] Secretaría de Finanzas y Planeación del Estado de Veracruz (SEFIPLAN) (2020). Estudios Regionales para la Planeación.

[13] Secretaría de Agricultura, Ganadería, Desarrollo Rural, Pesca y Alimentación (SAGARPA) (2007). Prospectiva del Subsector Agrícola del Estado de Veracruz.

[14] Goris M.G.A., Hartskeerl R.A. (2014). Leptospirosis serodiagnosis by the microscopic agglutination test. Curr. Protoc. Microbiol..

[15] Musso D., La Scola B. (2013). Laboratory diagnosis of leptospirosis: A challenge. J. Microbiol. Immunol. Infect..

[16] World Organisation for Animal Health (WOAH) (2021). Leptospirosis. Manual of Diagnostic Tests and Vaccines for Terrestrial Animals.

[17] Barros A.J.D., Hirakata V.N. (2003). Alternatives for logistic regression in cross-sectional studies: An empirical comparison of models that directly estimate the prevalence ratio. BMC Med. Res. Methodol..

[18] Romanowski T.N.A., Dias R.A., Heinemann M.B., Carvalho S.F., Silva T.A., Martins A.S., Caetano G.D.C., Ferreira Júnior Á., Santos J.P., Borsanelli A.C. (2023). Seroprevalence of Equine Leptospirosis in the State of Goiás, Brazil. Vet. Sci..

[19] Da Silva A.S., Jaguezeski A.M., Laber I.F., von Laer A.E., Lovato L.T., da Silva M.O., de Moura A.B. (2020). *Leptospira* spp. in horses in southern Brazil: Seroprevalence, infection risk factors, and influence on reproduction. Comp. Immunol. Microbiol. Infect. Dis..

[20] Arent Z.J., Kędzierska-Mieszkowska S. (2013). Seroprevalence study of leptospirosis in horses in northern Poland. Vet. Rec..

[21] Båverud V., Gunnarsson A., Olsson Engvall E., Franzén P., Egenvall A. (2009). *Leptospira* seroprevalence and associations between seropositivity, clinical disease and host factors in horses. Acta Vet. Scand..

[22] Simbizi V., Saulez M.N., Potts A., Lötter C., Gummow B. (2016). A study of leptospirosis in South African horses and associated risk factors. Prev. Vet. Med..

[23] Tsegay K., Potts A.D., Aklilu N., Lötter C., Gummow B. (2016). Circulating serovars of *Leptospira* in cart horses of central and southern Ethiopia and associated risk factors. Prev. Vet. Med..

[24] Siqueira C.C., Fraga D.B.M., Chagas-Junior A.D., Athanazio D.A., Silva M.M.N., Cerqueira R.B., McBride F.W.C., Pinna M.H., Ayres M.C.C. (2020). Seroprevalence and risk factors associated with equine leptospirosis in the metropolitan region of Salvador and Recôncavo Baiano region, Bahia state (NE Brazil). Trop. Anim. Health Prod..

[25] da Silva Martins A., Silva T.A., Heinemann M.B., Guedes I.B., Soares R.R., Dias R.A., Passos R.F., Braga R.M., dos Santos F.C.C., Borsanelli A.C. (2026). Seroprevalence of *Leptospira* in Lavradeiro horses, a semi-feral ecotype from the Northern Brazilian Amazon. Vet. Res. Commun..

[26] Tuemmers C., Quezada G., Morales R., Serri M. (2021). Seroprevalencia de *Leptospira* spp. en equinos de tiro de comunidades indígenas mapuche de la Región de La Araucanía, Chile. Rev. Chil. Infectol..

[27] Bolwell C.F., Rogers C.W., Benschop J., Collins-Emerson J.M., Adams B., Scarfe K.R., Gee E.K. (2020). Seroprevalence of *Leptospira* in Racehorses and Broodmares in New Zealand. Animals.

[28] McCreight K.A., Barbosa L.N., Odoi A., Reed P., Rajeev S. (2025). *Leptospira* seroprevalence in dogs, cats, and horses in Tennessee, USA. J. Vet. Diagn. Investig..

[29] Morais D.A., Nunes B.C., Barnabé N.N.C., Anjos D.M., Bezerra C.S., Costa D.F., Santos C.S.A.B., Azevedo S.S., Alves C.J. (2024). Spatial and seasonal distribution of *Leptospira* spp. seropositive horses in the Northeast region of Brazil. Prev. Vet. Med..

[30] Arent Z., Frizzell C., Gilmore C., Allen A., Ellis W.A. (2016). *Leptospira interrogans* serovars Bratislava and Muenchen animal infections: Implications for epidemiology and control. Vet. Microbiol..

[31] Vera E., Taddei S., Cavirani S., Schiavi J., Angelone M., Cabassi C.S., Schiano E., Quintavalla F. (2020). *Leptospira* Seroprevalence in Bardigiano Horses in Northern Italy. Animals.

[32] Fagre A.C., Mayo C.E., Pabilonia K.L., Landolt G.A. (2020). Seroprevalence of *Leptospira* spp. in Colorado equids and association with clinical disease. J. Vet. Diagn. Investig..

[33] Hamond C., Adam E.N., Stone N.E., LeCount K., Anderson T., Putz E.J., Camp P., Hicks J., Stuber T., van der Linden H. (2024). Identification of equine mares as reservoir hosts for pathogenic species of *Leptospira*. Front. Vet. Sci..

[34] Hartskeerl R.A., Goris M.G.A., Brem S., Meyer P., Kopp H., Gerhards H., Wollanke B. (2004). Classification of *Leptospira* from the eyes of horses suffering from recurrent uveitis. J. Vet. Med. B.

[35] Rocha T., Ellis W.A., Montgomery J., Gilmore C., Regalla J., Brem S. (2004). Microbiological and serological study of leptospirosis in horses at slaughter: First isolations. Res. Vet. Sci..

[36] Calderón J.C., Astudillo M., Romero M.H. (2019). Caracterización epidemiológica de la infección por *Leptospira* spp. en caballos de trabajo y en personas ocupacionalmente expuestas en seis unidades de la Policía Nacional de Colombia. Biomédica.

[37] Meny P., Iglesias T., Menéndez C., Quintero J., Ríos C., Ashfield N., Ferreira O., Mosca V., De Brun L., Ortiz G. (2022). Seroprevalence of anti-*Leptospira* antibodies in equines and associated workers—Isolation of *Leptospira interrogans* serogroup Canicola from equine urine. Zoonoses Public Health.

[38] Díaz E.A., Sáenz C., Guzmán D., Galecio J.S., Pearson T., Barragán V. (2026). *Leptospira* in Working Horses From Rural Ecuador: A Neglected Occupational Risk. J. Trop. Med..

